# The Effect of Integrated Lifestyle Intervention Incorporating Calorie‐Carbohydrate Restriction With or Without Time‐Restricted Feeding for Remission of Type 2 Diabetes (DIREM): A Single Blind Randomised Controlled Trial

**DOI:** 10.1002/edm2.70209

**Published:** 2026-04-11

**Authors:** Negin Badrooj, Alireza Esteghamati, Kurosh Djafarian, Mir Saeed Yekaninejad, Saba Mohammadpour, Vida Dankoob, Sahar Karimpour Reyhan, Mahsa Abbaszade, Sara Emamgholipour, Sakineh Shab‐Bidar

**Affiliations:** ^1^ Department of Community Nutrition, School of Nutritional Sciences and Dietetics Tehran University of Medical Sciences Tehran Iran; ^2^ Endocrinology and Metabolism Research Center (EMRC), Vali‐Asr Hospital Tehran University of Medical Sciences Tehran Iran; ^3^ Department of Clinical Nutrition, School of Nutritional Sciences and Dietetics Tehran University of Medical Sciences Tehran Iran; ^4^ Department of Epidemiology and Biostatistics, School of Public Health Tehran University of Medical Sciences Tehran Iran; ^5^ Department of Clinical Nutrition and Dietetics, Faculty of Nutrition Science and Food Technology Shahid Beheshti University of Medical Sciences Tehran Iran; ^6^ Department of Management & Health Economics, School of Public Health Tehran University of Medical Sciences Tehran Iran; ^7^ Sports Medicine Research Center Neuroscience Institute, Tehran University of Medical Sciences Tehran Iran; ^8^ Health Economics and Health Technology Assessment School of Health and Wellbeing, University of Glasgow Glasgow UK

**Keywords:** caloric restriction, carbohydrate restriction, intermittent fasting, remission, type 2 diabetes

## Abstract

**Aims:**

We conducted the diet and diabetes remission (DIREM) study to assess whether an integrated lifestyle intervention would lead to achieving remission in type 2 diabetes.

**Materials and Methods:**

Patients with type 2 diabetes were randomly assigned to calorie‐carbohydrate restriction (CCR) group, intermittent fasting with calorie‐carbohydrate restriction (IFCCR), or usual care group (control). The total study duration was 6 months, consisting of two phases: a 12‐week integrated lifestyle intervention (ILI) phase, followed by a 12‐week maintenance and structured monitoring (MSM) phase. The intervention was presented in the form of a structured behavioural model and also emphasised physical activity.

**Results:**

One hundred and twenty participants were randomly assigned to the study. Diabetes remission occurred in 9 (22.5%) of 40 participants in the CCR group (OR (CCR vs. Control) = 11.7, 95% CI: 1.4–98.3; *p* = 0.024), 12 (30.0%) of 40 participants in the IFCCR group (OR (IFCCR vs. Control) = 18.1, 95% CI: 2.2–151.0; *p* = 0.007) and 1 (2.5%) of 40 participants in the control group. The odds of remission were higher in the IFCCR group compared to the CCR group, but it was not significant (OR (IFCCR vs. CCR) = 1.5, 95% CI: 0.6–4.3; *p* = 0.4).

**Conclusions:**

Both calorie‐carbohydrate restriction alone and in combination with intermittent fasting significantly improved glycemic control and induced diabetes remission compared with the control group. No significant difference was found between the two interventions. Larger long‐term studies are needed to confirm these findings.

**Trial Registration:**

This trial was registered in the Iranian Registry of Clinical Trials (IRCT), IRCT20240418061519N1 (https://trialsearch.who.int/Trial2.aspx?TrialID=IRCT20240418061519N1)

## Graphical abstract

Diabetes remission is possible through weight loss. Behavioural lifestyle program is effective to achieve diabetes remission.

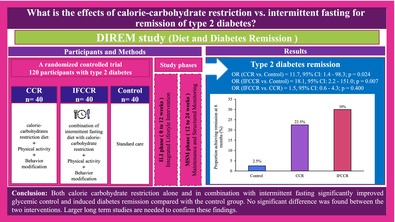

**Abbreviations**
CCR: calorie‐carbohydrates restriction
DIREM: diet and diabetes remission
IFCCR: intermittent fasting diet with calorie‐carbohydrate restriction
ILI phase: integrated lifestyle intervention phase
IMB model: information, motivation, and behavioural skill model
ITT: intention to treat analysis
MSM phase: maintenance and structured monitoring phase

## 1 Introduction

Type 2 diabetes is a critical global health crisis that, despite pharmacological advancements, long‐term complications persist even with optimised treatment [1, 2]. Various methods, such as extreme weight loss with bariatric surgery, calorie restriction, total replacement of diet with formula, and replacement of some meals with formula, have been shown to result in type 2 diabetes remission [3, 4, 5, 6, 7, 8]. In the DiRECT study, which involved a total diet replacement (TDR) intervention, diabetes remission was observed in 46% of the intervention group within 1 year. The DiRECT program sustained remissions at 2 years for 36% and at 5 years for 10% of people with type 2 diabetes [4, 5, 6]. The DIADEM‐I study, which investigated the intervention of formula‐based TDR, showed a 61% remission rate at 12‐month follow‐up [7]. These strategies operate through two key mechanisms: caloric restriction induces negative energy balance to reduce ectopic fat and improve insulin sensitivity [9] while carbohydrate restriction limits insulin‐mediated lipogenesis and enhances glycemic control [10, 11].

Emerging approaches like intermittent fasting (IF), particularly time‐restricted feeding (TRF), as the fasting window is shorter than other protocols, may amplify these effects by prolonging fasting periods to augment fat oxidation. TRF has higher compliance due to the shorter fasting period compared to other protocols and the lower risk of hypoglycemia in patients with diabetes [12]. Despite progress, critical gaps remain, such as most data on remission of type 2 diabetes being derived from formula‐based TDR [4, 5, 6, 7], which limits real‐world applicability and cost efficiency; the effectiveness of IF combined with calorie‐carbohydrate restriction compared with calorie‐carbohydrate restriction alone has not been tested, and long‐term adherence to structured food‐based interventions has been less well evaluated. It is essential to note, however, that while these studies and others use the term ‘remission’, its application remains a subject of debate. In our study, remission is operationally defined as achieving HbA1c < 6.5% without pharmacologic therapy for at least 3 months, which reflects short‐term, drug‐free glycemic control rather than permanent metabolic reversal.

To address these gaps, we conducted the diet and diabetes remission (DIREM) trial, comparing two food‐based dietary approaches, calorie‐carbohydrate restriction (CCR) and intermittent fasting plus calorie‐carbohydrate restriction (IFCCR), within a behavioural lifestyle program to achieve remission of type 2 diabetes.

## 2 Materials and Methods

### 2.1 Study Design

The DIREM study was a single‐blind, randomised, parallel‐group and controlled clinical trial conducted at a specialised diabetes clinic affiliated with Tehran University of Medical Sciences in Iran. Patients were recruited from May 2024 to June 2025. Ethical approval was obtained from the Tehran University of Medical Sciences Research Ethics Committee (IR.TUMS.SHARIATI.REC.1402.128). This study was registered in the Iranian Registry of Clinical Trials (IRCT) with the code number IRCT20240418061519N1. The protocol, including details of the study design, recruitment, intervention, and monitoring phases, and planned statistical analyses, has been published elsewhere [13].

### 2.2 Participants Recruitment and Sample Size Calculation

Participants were recruited from three outpatient endocrinology clinics and two tertiary care hospitals in Tehran, Iran. The sample size was calculated based on the findings from the Diabetes Remission Clinical Trial (DIRECT) [4]. With a type I error (*α*) of 0.05 and a type II error (*β*) of 0.15 (i.e., 85% statistical power), and accounting for an estimated 20% dropout rate, a total of 120 participants were enrolled in the study.

### 2.3 Selection Criteria

Eligible participants were aged 18–60 years, with less than 5 years duration of type 2 diabetes diagnosis. This cutoff was selected based on evidence from major diabetes remission studies, which have consistently shown that remission is most achievable in the early years following diagnosis, when residual beta‐cell function is better preserved [4, 7, 14, 15, 16]. Participants were eligible if they were taking at least one blood glucose‐lowering medication, had HbA1c more than 6.1% with the use of blood glucose‐lowering medication, and had a body mass index (BMI) of 27–40 kg/m^2^. This range of BMI was selected based on clinical guidelines and alignment with major diabetes remission trials [4, 7, 17, 18, 19]. Exclusion criteria included use of insulin or GLP‐1 receptor agonists, severe hyperglycemia (HbA1c ≥ 10%), recent significant weight loss and any major comorbidities. Patients receiving insulin therapy were excluded, as insulin use reflects more advanced β‐cell dysfunction and conflicts with the mechanistic basis of diabetes remission, which requires sufficient residual insulin secretory capacity [16].

### 2.4 Randomisation and Masking

Participants were randomly allocated to one of our three groups: the calorie‐carbohydrate restriction group (CCR), the intermittent fasting with calorie‐carbohydrate restriction group (IFCCR), or the guideline‐based usual care group (control). Randomisation was done using the Rand software and the stratified (gender) permuted block randomisation method. All assessors remained unaware of subjects' allocation to study groups. Due to the nature of the intervention, participants and intervention providers were aware of group allocation.

### 2.5 Procedures

Participants first completed a 2‐week run‐in period during which informed consent was obtained and baseline dietary information was recorded. After this period, they were randomly assigned to one of three groups: CCR, IFCCR, or control groups. During the 12‐week integrated lifestyle intervention (ILI) phase, participants in the intervention groups received a structured diabetes self‐management program based on the IMB model, delivered through group education, individual counselling, and remote support [20, 21]. Dietary prescriptions differed between groups, with the CCR group following a diet with 1000–1200 kcal energy, restricted to 40% carbohydrates, with a protein and fat distribution of 30% and 30%, respectively, whereas the IFCCR group followed the same diet within a defined 16:8 time‐restricted eating window [22]. Both intervention groups received recommendations for moderate‐intensity physical activity [23]. Antidiabetic medication doses were adjusted and documented by the study endocrinologist according to participants' glycemic control. Given the carbohydrate restriction implemented in both intervention groups, SGLT2 inhibitors were initially reduced or discontinued to minimise the risk of adverse events. During the intervention, medication adjustments were guided by the average of all self‐monitored blood glucose measurements recorded over a 2‐week period. If the 2‐week average of self‐monitored blood glucose levels was consistently below the clinically defined safety threshold for deprescribing, medications were reduced or discontinued in a stepwise manner: first sulfonylureas, followed by thiazolidinediones, then alpha‐glucosidase inhibitors, and finally metformin. Participants also attended scheduled counselling sessions and regularly reported their blood glucose levels and weight via phone or social media. The control group received standard guideline‐based dietary, physical activity, and diabetes education [24].

Following completion of the intensive intervention, participants entered a 12‐week maintenance and structured monitoring (MSM) phase designed to support weight‐loss maintenance. During this phase, they were advised to continue a food‐based maintenance diet, attended monthly group sessions, and continued remote reporting of blood glucose and weight for monitoring and safety. In this study, a ‘food‐based’ diet refers to a structured dietary prescription composed of conventional whole foods prepared by participants, rather than commercially prepared formula products or total diet replacement (TDR) meal substitutes. Participants in the control group continued receiving routine standard care during this period [24].

### 2.6 Outcomes

The primary outcome of the study was remission in type 2 diabetes, defined as achieving an HbA1c level below 6.5% without glucose‐lowering medication for at least 3 months. Secondary outcomes included additional glycemic markers, lipid profile parameters, liver enzymes, quality of life, and the number of antidiabetic medications used. Insulin resistance was assessed using the homeostatic model assessment of insulin resistance (HOMA‐IR), calculated as: [fasting insulin (μU/mL) × fasting glucose (mmol/L)]/22.5 [25]. Insulin sensitivity was evaluated using the quantitative insulin sensitivity check index (QUICKI), calculated as: 1/[log (fasting insulin in μU/mL) + log (fasting glucose in mg/dL)]. For QUICKI calculation, glucose values were converted from mmol/L to mg/dL by multiplying by 18.01 [26]. All outcomes were assessed at baseline, 3 and 6 months.

To characterise participants, demographic information, medical history, smoking status, and socioeconomic indicators were collected using standardised questionnaires. Blood pressure was measured twice and averaged. Physical activity was assessed with the International Physical Activity Questionnaire [27], and dietary intake was monitored through repeated 3‐day food records analysed using specialised nutrition software. Anthropometric measurements—including body weight, height, waist circumference, and body composition—were obtained at each study phase by a trained examiner using standardised procedures. Bioelectrical impedance analysis (BIA) (TANITA BC‐418) was used to measure body composition indices including body fat mass and fat‐free mass. All BIA measurements were performed under standardised conditions: between 7:00 and 9:00 AM, with participants in a supine position for at least 10 min, after voiding and with normal hydration. Participants were asked to avoid intense physical activity prior to each measurement.

Quality of life was evaluated with the EQ‐5D‐5L instrument, which captures key dimensions of health status across multiple levels of severity [28, 29]. Biochemical assessments included fasting blood glucose, insulin, HbA1c, lipid profile, liver enzymes, and renal function indices, all measured from fasting blood samples collected at each assessment point.

Adherence to the interventions was supported through structured behavioural counselling and frequent monitoring. Participants received regular in‐person sessions with nutritionists and maintained ongoing contact through remote reporting of blood glucose and body weight. These follow‐ups allowed timely reinforcement of dietary recommendations and, for participants in the time‐restricted eating group, verification of adherence to the prescribed eating window.

### 2.7 Statistical Analysis

All analyses were conducted based on the intention‐to‐treat (ITT) principles. Sensitivity analyses were performed using per‐protocol approaches. For remission outcomes, dropouts were conservatively classified as no remission. A Linear Mixed Model (LMM) was used to compare the average changes of continuous outcomes. The effects of treatment method, time, and the interaction effect of group and time were investigated by LMM, controlling for confounding variables (age, gender, baseline BMI, and baseline values as fixed effects). Adjusted means were reported according to LMM. Multiple logistic regression analysis was used to compare the odds of remission in three study groups, adjusting for age, sex, and BMI at baseline. All analyses were conducted on STATA 17 (Stata Corp, College Station, TX, USA). In all analyses, a *p*‐value < 0.05 was considered statistically significant, and all statistical tests were two‐sided.

## 3 Results

**FIGURE 1:**
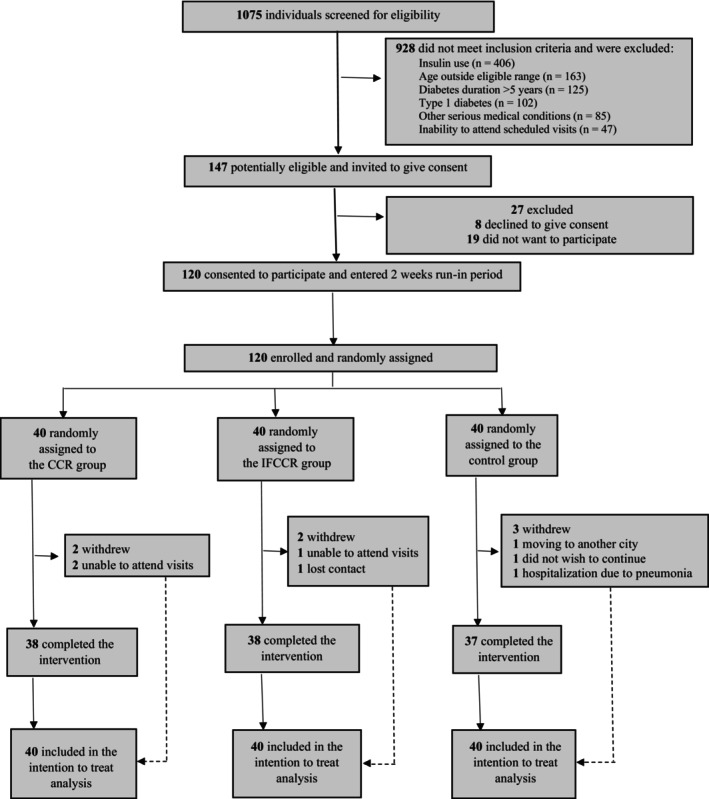
CONSORT flow diagram. CCR group, calorie‐carbohydrate restriction group; IFCCR group, intermittent fasting with calorie‐carbohydrate restriction group.

A total of 1075 potential participants were identified and were screened for eligibility (Figure 1). One hundred and forty‐seven (13.7%) of these individuals met the criteria for inclusion in the study. 120 (81.6%) of these 147 individuals provided consent to participate in the study and entered a 2‐week run‐in period. One hundred and twenty participants were enrolled and randomly assigned to the CCR group (*n* = 40), the IFCCR group (*n* = 40) or the control group (*n* = 40). A total of 120 participants were included in the final intention‐to‐treat analysis.

Baseline characteristics of participants are shown in Table 1. Mean age was 50.0 years (SD = 7.7) and BMI was 31.1 kg/m^2^ (SD = 3.3). Mean diabetes duration was 3.1 years (SD = 2.2). The three groups were well‐balanced at randomisation across key demographic, clinical and metabolic variables and also socioeconomic characteristics. Mean HbA1c was 7.2% (SD = 1.1; 57.2 mmol/mol [SD = 9.9]). At baseline, metformin was used by all participants (100%). Among other antihyperglycemic medications, DPP‐4 inhibitors were the most commonly used (39.2%), followed by SGLT2 inhibitors (38.3%) and sulfonylureas (35.0%). Thiazolidinediones were used by a small proportion of participants (4.2%). 109 (90.8%) of all participants were taking statins. Antihypertensive medications were used by 42 participants, representing 35% of the study population. The mean of total energy intake was 2326.4 kcal/day (SD = 314.4) and the mean percentage of carbohydrate, protein, and total fat was 62.5% (SD = 2.5), 13.5% (SD = 2.1), and 24.4% (SD = 1.9), respectively.

**TABLE 1:** Baseline characteristics.

Variables	CCR group (*n* = 40)	IFCCR group (*n* = 40)	Control group (*n* = 40)
Female	20 (50.0%)	20 (50.0%)	20 (50.0%)
Male	20 (50.0%)	20 (50.0%)	20 (50.0%)
Age, years	49.4 (6.3)	49.0 (7.6)	51.6 (7.0)
Education status			
High school or less	3 (7.5%)	8 (20.0%)	6 (15.0%)
Diploma	10 (25.0%)	13 (32.5%)	11 (27.5%)
University degree or higher	27 (67.5%)	19 (47.5%)	23 (57.5%)
Employment status			
Employed	26 (65.0%)	23 (57.5%)	20 (50.0%)
Unemployed	9 (22.5%)	11 (27.5%)	12 (30.0%)
Retired	5 (12.5%)	6 (15.0%)	8 (20.0%)
Bodyweight, kg	86.0 (9.5)	86.8 (9.5)	86.0 (12.0)
BMI, kg/m^2^	31.0 (2.5)	31.5 (3.2)	30.7 (2.5)
Waist circumference, cm	106.4 (8.9)	106.6 (8.9)	108.9 (8.2)
Fat mass, kg	29.4 (8.2)	30.1 (8.9)	28.4 (5.7)
Fat free mass, kg	56.3 (8.9)	56.7 (10.8)	57.5 (12.9)
Duration of diabetes, years	3.0 (1.9)	2.8 (1.9)	3.4 (1.9)
Number of diabetes medications	4.1 (1.9)	3.9 (1.9)	3.8 (1.9)
Type of diabetes medication			
Metformin	40 (100%)	40 (100%)	40 (100%)
Sulfonylurea	16 (40.0%)	15 (37.5%)	11 (27.5%)
DPP‐4 inhibitor	14 (40.0%)	18 (45.0%)	15 (37.5%)
Thiazolidinedione	2 (5.0%)	1 (2.5%)	2 (5.0%)
SGLT2 inhibitor	16 (40.0%)	14 (35.0%)	16 (40.0%)
HbA1c, %	7.0 (0.6)	7.3 (0.6)	7.4 (1.3)
HbA1c, mmol/mol	55.1 (7.6)	58.0 (9.5)	58.6 (11.4)
FBG, mmol/L	7.2 (1.3)	7.5 (1.9)	7.5 (1.9)
QUCKI	0.32 (0.02)	0.31 (0.02)	0.32 (0.03)
HOMAIR	4.0 (1.9)	4.5 (1.9)	4.0 (2.5)
Systolic blood pressure, mm Hg	132.4 (12.6)	128.0 (11.4)	126.4 (8.9)
Diastolic blood pressure, mm Hg	87.0 (8.9)	86.4 (9.5)	84.6 (7.6)
Prescribed antihypertensive medication	14 (35.0%)	15 (37.5%)	13 (32.5%)
Prescribed statins	38 (95.0%)	34 (85.0%)	37 (92.5%)
Total cholesterol, mmol/L	4.0 (0.6)	3.8 (0.6)	4.0 (0.6)
HDL cholesterol, mmol/L	1.1 (0.3)	1.0 (0.3)	1.1 (0.3)
LDL cholesterol, mmol/L	2.3 (0.6)	2.1 (0.6)	2.1 (0.6)
Triglycerides, mmol/L	1.7 (1.2–2.1)	1.6 (1.2–2.2)	1.2 (0.9–2.0)
AST, U/L	29.6 (6.3)	29.2 (9.5)	26.5 (6.3)
ALT, U/L	36.1 (10.8)	37.6 (17.1)	32.6 (9.5)
eGFR, mL/min per 1.73 m^2^ ^a^	82.0 (11.4)	81.3 (11.4)	83.8 (9.5)
Current smoker	10 (25.0%)	8 (20.0%)	7 (17.5%)
Physical activity, MET.min/week	650.2 (125.2)	636.2 (129.7)	613.4 (120.2)
EQ‐5D scale score	0.54 (0.2)	0.55 (0.2)	0.54 (0.2)
Total energy intake, kcal/day	2352.5 (297.3)	2271.1 (302.3)	2355.5 (342.2)
Carbohydrate intake, g/day	371.2 (56.9)	360.3 (56.3)	362.0 (64.5)
Protein intake, g/day	78.6 (17.7)	75.6 (16.4)	81.0 (10.8)
Total fat intake, g/day	62.8 (5.7)	60.8 (6.3)	64.9 (8.9)

*Note:* Data are *n* (%), mean (SD) and median (IQR).

Abbreviations: ALT, alanine transaminase; AST, aspartate aminotransferase; BMI, body mass index; eGFR, estimated glomerular filtration rate; EQ‐5D, EuroQol 5 dimensions; FBG, fasting blood glucose; HbA1c, glycated haemoglobin; HDL, high density lipoprotein; HOMAIR, homeostatic model assessment for insulin resistance; LDL, low density lipoprotein; QUICKI, quantitative insulin sensitivity index.

^a^ According to the modification of diet in renal disease study equation.

**FIGURE 2:**
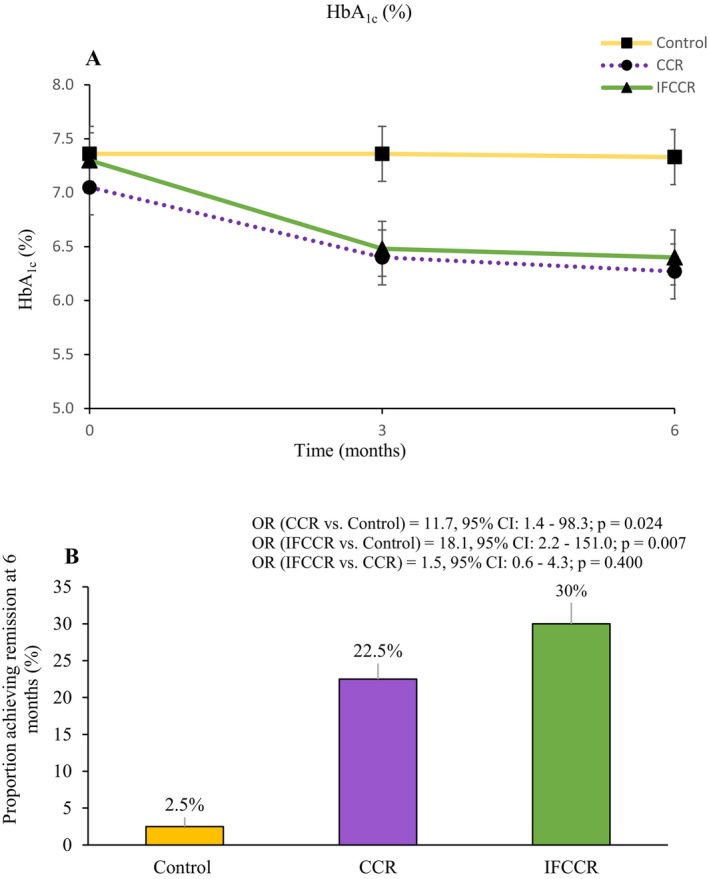
Glycaemia outcomes (A) changes in glycated haemoglobin (HbA1C) during the study periods (B) Proportion of participants who had diabetes remission (defined as HbA1c < 6.5% [< 48 mmol/mol] and no medications for 3 months).

The mean HbA1c in the CCR group at 3 months was 6.40% (SE = 0.13), which was decreased from baseline, and this decreasing trend was maintained at 6 months, but with a lower slope (6.27% [SE = 0.13]). The IFCCR group had a similar decreasing trend to the CCR group at 3 and 6 months (6.48% [SE = 0.13] and 6.40% [SE = 0.13], respectively), although there was no significant difference at the two time points between the two intervention groups. The mean HbA1c of both the CCR group and the IFCCR group was significantly lower at two time points compared with the control group (*p* < 0.001) (Table 2; Figure 2A).

**TABLE 2:** Key outcome variables at 3‐ and 6‐month assessments.

Variables	Baseline			3 months						6 months					
	Control	CCR	IFCCR	Control	CCR		IFCCR			Control	CCR		IFCCR		
	Mean (SE)	Mean (SE)	Mean (SE)	Mean (SE)	Mean (SE)	p*	Mean (SE)	p**	p***	Mean (SE)	Mean (SE)	p*	Mean (SE)	p**	p***
HbA1c, %	7.36 (0.13)	7.05 (0.13)	7.30 (0.13)	7.36 (0.13)	6.40 (0.13)	< 0.001	6.48 (0.13)	< 0.001	0.120	7.33 (0.13)	6.27 (0.13)	< 0.001	6.40 (0.13)	< 0.001	0.297
HbA1c, mmol/mol	58.57 (1.46)	55.18 (1.45)	57.93 (1.45)	58.59 (1.48)	48.12 (1.46)	< 0.001	48.95 (1.46)	< 0.001	0.120	58.23 (1.48)	46.63 (1.46)	< 0.001	48.09 (1.46)	< 0.001	0.297
FBG, mmol/L	7.51 (0.23)	7.23 (0.22)	7.48 (0.23)	7.53 (0.23)	6.13 (0.23)	< 0.001	6.16 (0.23)	< 0.001	0.313	7.44 (0.23)	5.66 (0.23)	< 0.001	5.92 (0.23)	< 0.001	0.924
QUICKI	0.32 (0.004)	0.32 (0.003)	0.31 (0.004)	0.31 (0.004)	0.34 (0.004)	< 0.001	0.33 (0.004)	< 0.001	0.748	0.31 (0.004)	0.34 (0.004)	< 0.001	0.34 (0.004)	< 0.001	0.495
HOMAIR	4.09 (0.31)	3.94 (0.30)	4.50 (0.31)	4.61 (0.31)	2.60 (0.31)	< 0.001	2.80 (0.31)	< 0.001	0.151	4.95 (0.31)	2.28 (0.31)	< 0.001	2.41 (0.31)	< 0.001	0.086
Weight, kg	87.43 (1.02)	86.11 (1.01)	85.52 (1.02)	87.74 (1.02)	81.81 (1.01)	< 0.001	80.24 (1.02)	< 0.001	0.060	87.94 (1.02)	81.01 (1.01)	< 0.001	79.47 (1.02)	< 0.001	0.066
WC, cm	109.95 (0.97)	106.66 (0.96)	105.59 (0.97)	111.04 (0.98)	102.04 (0.97)	< 0.001	101.10 (0.98)	< 0.001	0.910	111.21 (0.99)	100.54 (0.97)	< 0.001	98.29 (0.98)	< 0.001	0.276
Fat mass, kg	29.15 (0.63)	29.61 (0.63)	29.36 (0.63)	29.49 (0.64)	25.19 (0.63)	< 0.001	23.33 (0.64)	< 0.001	0.007	29.73 (0.64)	24.11 (0.63)	< 0.001	22.32 (0.64)	< 0.001	0.009
Fat free mass, kg	58.18 (0.93)	56.21 (0.92)	56.17 (0.93)	58.08 (0.93)	56.58 (0.92)	0.070	56.91 (0.93)	0.001	0.151	58.02 (0.93)	56.89 (0.92)	0.001	57.02 (0.93)	< 0.001	0.512
TC, mmol/L	3.97 (0.12)	4.02 (0.12)	3.83 (0.12)	4.21 (0.12)	3.60 (0.12)	< 0.001	3.50 (0.12)	< 0.001	0.399	4.43 (0.12)	3.49 (0.12)	< 0.001	3.36 (0.12)	< 0.001	0.540
HDL, mmol/L	1.12 (0.04)	1.06 (0.04)	1.05 (0.04)	1.04 (0.04)	1.15 (0.04)	< 0.001	1.14 (0.04)	< 0.001	0.875	1.00 (0.04)	1.21 (0.04)	< 0.001	1.18 (0.04)	< 0.001	0.631
LDL, mmol/L	2.17 (0.11)	2.24 (0.11)	2.05 (0.11)	2.52 (0.11)	1.93 (0.11)	< 0.001	1.78 (0.11)	< 0.001	0.625	2.77 (0.11)	1.81 (0.11)	< 0.001	1.69 (0.11)	< 0.001	0.470
Triglyceride, mmol/L	1.67 (0.12)	1.76 (0.12)	1.74 (0.12)	1.86 (0.12)	1.40 (0.12)	< 0.001	1.31 (0.12)	< 0.001	0.500	1.92 (0.12)	1.38 (0.12)	< 0.001	1.24 (0.12)	< 0.001	0.261
AST (U/L)	26.66 (1.03)	29.54 (1.02)	29.05 (1.02)	28.31 (1.04)	23.34 (1.03)	< 0.001	23.07 (1.03)	< 0.001	0.828	29.79 (1.04)	20.92 (1.03)	< 0.001	21.12 (1.03)	< 0.001	0.489
ALT (U/L)	33.23 (1.59)	35.91 (1.58)	37.06 (1.59)	34.50 (1.63)	27.66 (1.60)	< 0.001	27.88 (1.61)	< 0.001	0.615	36.72 (1.63)	24.53 (1.60)	< 0.001	24.88 (1.61)	< 0.001	0.667
Number of diabetes medications	3.74 (0.29)	4.11 (0.29)	3.92 (0.29)	4.17 (0.29)	2.60 (0.29)	< 0.001	2.39 (0.29)	< 0.001	0.915	4.03 (0.29)	2.60 (0.29)	< 0.001	2.39 (0.29)	< 0.001	0.915
Systolic BP, mmHg	126.28 (1.41)	132.61 (1.40)	128.00 (1.41)	127.19 (1.44)	122.74 (1.42)	< 0.001	120.66 (1.42)	< 0.001	0.088	128.44 (1.44)	119.16 (1.42)	< 0.001	116.93 (1.42)	< 0.001	0.110
Diastolic BP, mmHg	84.70 (1.09)	86.98 (1.08)	86.31 (1.09)	86.60 (1.11)	79.12 (1.10)	< 0.001	78.78 (1.10)	< 0.001	0.791	87.58 (1.11)	76.41 (1.10)	< 0.001	75.07 (1.10)	< 0.001	0.589
Quality of life^a^	0.547 (0.02)	0.534 (0.02)	0.542 (0.02)	0.582 (0.03)	0.794 (0.03)	< 0.001	0.791 (0.03)	< 0.001	0.703	0.591 (0.03)	0.847 (0.03)	< 0.001	0.831 (0.03)	< 0.001	0.407
Information score^b^	2.99 (0.20)	2.76 (0.20)	2.88 (0.20)	3.51 (0.21)	4.54 (0.21)	< 0.001	4.65 (0.21)	< 0.001	0.951	3.51 (0.21)	4.57 (0.21)	0.001	4.62 (0.21)	0.002	0.809
Motivation score^b^	26.99 (0.69)	24.25 (0.69)	25.57 (0.69)	23.93 (0.71)	34.40 (0.70)	< 0.001	36.42 (0.70)	< 0.001	0.400	23.63 (0.71)	33.77 (0.70)	< 0.001	35.68 (0.70)	< 0.001	0.474
Behavioural skills score^b^	4.07 (0.29)	3.96 (0.29)	4.08 (0.29)	4.11 (0.30)	6.66 (0.30)	< 0.001	7.02 (0.30)	< 0.001	0.572	4.11 (0.30)	6.29 (0.30)	< 0.001	6.73 (0.30)	< 0.001	0.455
Total score^b^	33.54 (0.94)	30.93 (0.93)	32.45 (0.93)	30.53 (0.95)	45.59 (0.94)	< 0.001	48.01 (0.94)	< 0.001	0.388	30.36 (0.96)	44.35 (0.94)	< 0.001	46.96 (0.94)	< 0.001	0.298

*Note:* Adjusted means for outcome variables controlling for age, gender, baseline BMI, and baseline values as fixed effects. p*: *p* value comparing the mean change of response variables in CCR group vs. control group. p**: *p* value comparing the mean change of response variables in IFCCR group vs. control group. p***: *p* value comparing the mean change of response variables in IFCCR group vs. CCR group.

Abbreviations: ALT, alanine transaminase; AST, aspartate aminotransferase; DBP, diastolic blood pressure; FBG, fasting blood glucose; HbA1c, glycated haemoglobin; HDL, high density lipoprotein; HOMAIR, homeostatic model assessment for insulin resistance; LDL, low density lipoprotein; QUICKI, quantitative insulin sensitivity index; SBP, systolic blood pressure; TC, total cholesterol; WC, waist circumstance.

^a^ As measured by the EuroQol 5 dimensions scale.

^b^ Based on Information, motivation and behavioural skills (IMB) model.

Remission occurred in 9 (22.5%) of 40 participants in the CCR group (OR [CCR vs. Control] = 11.7, 95% CI: 1.4–98.3; *p* = 0.024), 12 (30.0%) of 40 participants in the IFCCR group (OR [IFCCR vs. Control] = 18.1, 95% CI: 2.2–151.0; *p* = 0.007) and 1 (2.5%) of 40 participants in the control group. The odds of remission were higher in the IFCCR group compared to the CCR group, but it was not significant (OR [IFCCR vs. CCR] = 1.5, 95% CI: 0.6–4.3; *p* = 0.400) (Figure 2B). As shown in Table 3, both intervention groups achieved a significantly higher remission proportion than the control group based on risk difference (RD). The RD for CCR versus control was 20.0% (95% CI: 6.18%–33.81%; *p* = 0.007), and for IFCCR versus control was 27.5% (95% CI: 12.5%–42.5%; *p* < 0.001). The RD between IFCCR and CCR was 7.5% (95% CI: 0%–26.71%; *p* = 0.424), indicating no statistically significant difference between the two active interventions. The number of diabetes medications was reduced in 67.5% (27 of 40) of participants in the CCR group and 70% (28 of 40) of participants in the IFCCR group at 6 months.

**TABLE 3:** Remission rates and risk differences (RD) between study groups.

	Control (*n* = 40)	CCR (*n* = 40)	IFCCR (*n* = 40)
Remission, *n* (%)	1 (2.5)	9 (22.5)	12 (30.0)

	RD (95% CI)	*p*
CCR vs. control	20% (6.18–33.81)	0.007
IFCCR vs. control	27.5% (12.5–42.5)	< 0.001
IFCCR vs. CCR	7.5% (0–26.71)	0.424

**FIGURE 3:**
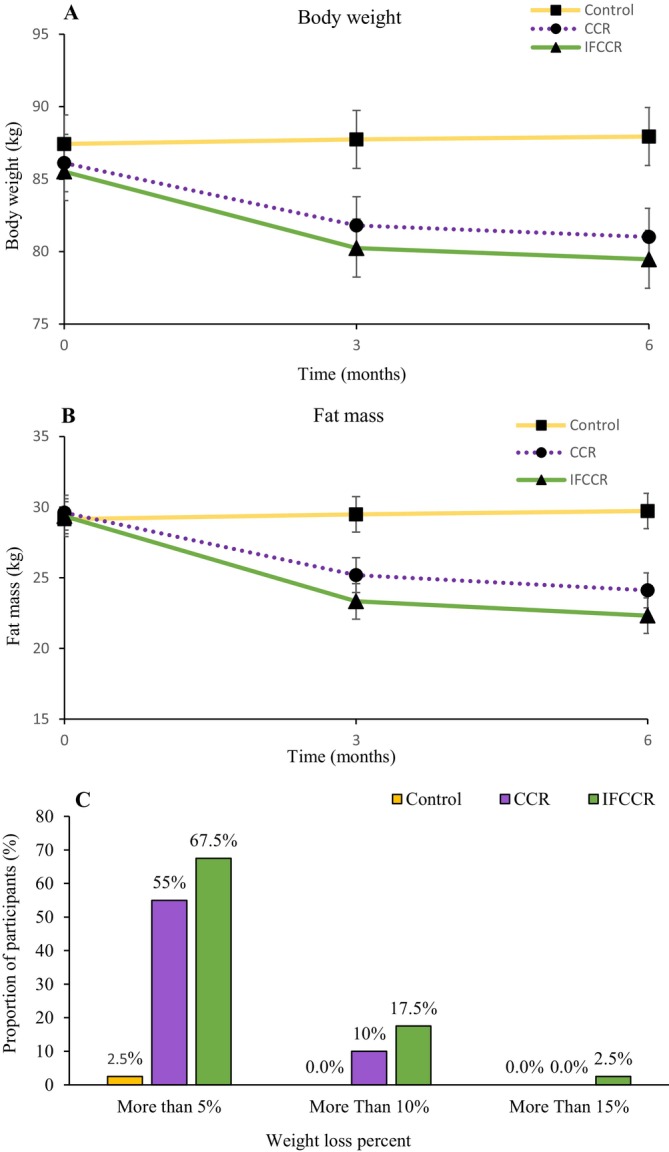
Weight and body composition. Bodyweight (A) and fat mass (B) during the study. (C) Proportion of participants achieving weight loss targets over 6 months.

In both the CCR groups and the IFCCR group, the greatest reduction in weight occurred at 3 months (81.81 [SE = 1.01] and 80.24 [SE: 1.02], respectively), followed by a slower but continued reduction in weight at 6 months. The mean weight of both the CCR group and the IFCCR group was significantly lower at two time points compared with the control group (*p* < 0.001). There was a borderline significant difference at both time points between the two intervention groups (Table 2; Figure 3A).

The mean fat mass in both intervention groups decreased from baseline at 3 months, with the mean fat mass in the IFCCR group being lower than that in the CCR group (IFCCR = 23.33 [SE = 0.64] vs. CCR = 25.19 [SE = 0.63]), which was a significant difference in both groups compared to the control group (*p* < 0.001) and also between the CCR group and the IFCCR group (*p* = 0.007). This decreasing trend for fat mass was maintained at 6 months, but with a lower slope and also with a significant difference between the two intervention groups compared to the control group (*p* < 0.001). Also, the difference between the CCR and the IFCCR groups remained significant at 6 months (*p* = 0.009) (Table 2; Figure 3B).

**FIGURE 4:**
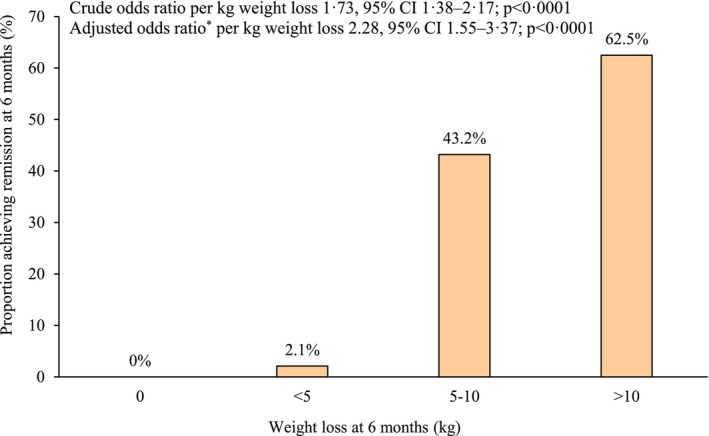
Remission of diabetes, in relation to weight loss achieved at 6 months (groups combined). *Based on multivariable logistic regression model adjusting for sex, age, baseline BMI, baseline HbA1c, and intervention group.

In the IFCCR group, 67.5% (27 of 40) and in the CCR group, 55% (22 of 40) of participants had weight loss of more than 5%. When targeting more than 5% weight loss at 6 months, participants in the IFCCR group had odds of 1.64 (95% CI: 0.65–4.12) compared with the CCR group. In the IFCCR group, 17.5% (7 of 40), and in the CCR group, 10% (4 of 40) of participants had weight loss of more than 10%. When targeting more than 10% weight loss at 6 months, participants in the IFCCR group had odds of 1.94 (95% CI: 0.51–7.35) compared with the CCR group, although the obtained results were not significant (Figure 3C). Remission varied with weight loss in the whole study population, with achievement by no participant who did not lose weight at 6 months, one (2.1%) of 47 participants who maintained 0–5 kg weight loss, 16 (43.2%) of 37 participants with 5–10 kg loss and 5 (62.5%) of 8 participants with 10–15 kg loss. Logistic regression analysis showed that each 1 kg of weight loss was associated with a 1.73‐fold increase in the odds of remission (OR = 1.73, 95% CI: 1.38–2.17; *p* < 0.001) (Figure 4). Also, multivariable logistic regression analysis was performed to adjust for potential confounders including sex, age, baseline BMI, baseline HbA1c and intervention group. The results showed that each 1 kg of weight loss was independently associated with a 2.28‐fold increase in the odds of remission (OR = 2.28, 95% CI: 1.55–3.37; *p* < 0.001) (Figure 4).

Mean quality of life scores, measured by use of the EQ‐5D‐5L index value according to the five dimensions of the questionnaire, are reported in Table 2. Also, the mean visual analogue scale of EQ‐5D was increased from baseline in both intervention groups (CCR: 57.22 [SE = 1.68] and IFCCR: 57.80 [SE = 1.69]) at 3 months (CCR: 78.99 [SE = 1.71] and IFCCR: 78.64 [SE = 1.71]) and at 6 months (CCR: 83.28 [SE = 1.71] and IFCCR: 82.90 [SE = 1.71]). Mean visual scale was also increased in the control group from baseline (59.98 [SE = 1.70]) at two time points (3 months: 63.97 [SE = 1.73] and 6 months: 64.03 [SE = 1.74]), but the difference was significant between the control group compared to both intervention groups (*p* < 0.001). Although the differences between the two intervention groups were not significant. Compared to baseline values (CCR: 650.2 [SE = 125.2], IFCCR: 636.2 [SE = 129.7]), mean physical activity (MET.min/week) increased in both intervention groups at 3 months (CCR: 1058.61 ± 27.58 and IFCCR: 1077.01 ± 27.68) and remained higher at 6 months (CCR: 1026.84 ± 27.58 and IFCCR: 1048.75 ± 27.68), with a significant difference compared to the control group (*p* < 0.001).

Adherence was assessed through three methods: completion of three‐day food records, attendance at individual/group sessions, and regular phone/text contact with the research team. The rate of regular food record completion was significantly higher in the IFCCR group (90.0% vs. 72.5% in CCR, *p* = 0.045). Overall, 81.3% of participants regularly attended sessions and 86.3% maintained regular contact with the research team.

For sensitivity analyses, all analyses were performed based on a per‐protocol approach, and the results were similar to the intention‐to‐treat analysis (Tables S1 and S2, Figures S1 and S2).

### 3.1 Adverse Events

There were no serious adverse events reported in the intervention groups. In the CCR group, one participant experienced mild hypoglycemia. None of the patients experienced gastrointestinal problems.

## 4 Discussion

In this randomised controlled trial, both the calorie‐carbohydrate restriction and intermittent fasting interventions significantly improved glycemic control and metabolic health in participants with type 2 diabetes. At 6 months, HbA1c levels decreased markedly in both intervention groups (CCR: 6.27%; IFCCR: 6.40%) compared to controls, with remission rates of 22.5% (CCR) and 30% (IFCCR)—significantly higher than the control group (2.5%). Weight, fat mass, lipid profiles, and insulin sensitivity (QUICKI, HOMA‐IR) also improved substantially in both intervention groups, with greater fat mass loss observed in the IFCCR group. The remission rate in the IFCCR group was 1.5 times that of the CCR group; however, this difference was not statistically significant, and the wide confidence interval suggests limited precision. Given the sparse event data, these results should be interpreted cautiously.

The significant difference observed in remission rate in the intervention groups of our study compared to the control group was aligned with prior trials demonstrating that dietary interventions can induce remission in type 2 diabetes, particularly when weight loss is achieved. For instance, the DiRECT study found a 46% remission rate compared to 4% in the control group at 12 months with a very low‐calorie diet (VLCD) and intensive lifestyle intervention, though their protocol involved stricter energy restriction [4]. Unlike formula‐based total diet replacement interventions, our approach relied exclusively on conventional foods, enhancing real‐world feasibility and cultural adaptability. The strong observed remission odds versus controls in our study nonetheless underscore the potential of structured dietary interventions as a scalable therapy for diabetes remission. Although the IFCCR group had numerically higher remission odds than the CCR group (OR = 1.5), this difference was not statistically significant, suggesting that the added benefit of intermittent fasting may be modest in this context. The remission observed in our trial strongly supports the ‘twin cycle’ hypothesis, whereby weight loss—particularly reduction in ectopic fat in liver and pancreas—improves insulin sensitivity and restores β‐cell function [16]. Since dietary carbohydrates and insulin have been shown to play an important role in the pathological accumulation of fat in the body, carbohydrate restriction is effective in type 2 diabetes remission [30, 31]. In the results, we observed that most of the achieved weight loss was related to the fat mass, and fat‐free mass was maintained. Notably, the IFCCR group had greater fat mass reduction than the CCR group at both time points, a finding echoed in a study showing that TRF and concurrent exercise training act as a dietary strategy to reduce fat mass and increase lean mass [32].

Contrary to our results, a very low‐calorie diet during 3 days of the week (3:4 intermittent fasting) reported no additional fat loss benefit over CCR, possibly due to compensatory eating during feeding days [33]. In our study, the diet prescribed to the intervention groups had a high protein percentage, which could lead to greater satiety, increased energy expenditure, and prevent the loss of lean mass [34]. Combining this high‐protein diet with increased physical activity, which emphasised resistance exercise alongside aerobic exercise, led to this result. A modest increase in fat‐free mass was observed in the intervention groups despite substantial weight loss. Although both dietary arms emphasised relatively higher protein intake and were accompanied by increased physical activity—factors known to attenuate fat‐free mass loss during caloric restriction—the magnitude of fat‐free mass gain (approximately 1–2 kg) should be interpreted cautiously. Body composition was assessed using bioelectrical impedance analysis, which is sensitive to hydration status and shifts in total body water. Given the carbohydrate‐restricted nature of the interventions, changes in glycogen stores and associated fluid balance may have influenced fat‐free mass estimates. Therefore, the observed increase in fat‐free mass may partly reflect methodological characteristics of bioelectrical impedance analysis rather than true anabolic gain. Future studies using more precise methods such as dual‐energy X‐ray absorptiometry are warranted to confirm these findings.

The success of our food‐based intervention in achieving these clinically meaningful remission rates can be attributed to several synergistic factors beyond simple calorie restriction. These outcomes likely resulted from the combined effects of carbohydrate restriction reducing glucotoxicity and lipogenesis, a high‐protein diet enhancing satiety and preserving fat‐free mass, structured aerobic and resistance exercise improving insulin sensitivity and fat loss, and strong behavioural support that boosted adherence.

### 4.1 Clinical Implications

From a clinical perspective, both dietary approaches—calorie‐carbohydrate restriction alone and in combination with intermittent fasting—were effective in improving glycemic control and leading to diabetes remission compared to usual care. Although the IFCCR group showed a numerically higher remission rate and greater fat mass reduction at both time points, these differences were not statistically significant and should be viewed with caution. The small number of remission events, particularly in the control group, resulted in wide confidence intervals and limited precision. Although this low rate is clinically expected given the natural history of diabetes without intervention, it presents statistical challenges and requires cautious interpretation of the effect estimates. In practice, both approaches are valid options for motivated patients. The choice may depend on individual preferences and lifestyle. Either way, these dietary changes should be part of a broader lifestyle program that includes regular physical activity and self‐management support. Given the preliminary nature of these findings, larger and longer studies are still needed to clarify whether one approach offers meaningful advantages over the other and to better understand which patients might benefit most from each. Beyond the clinical outcomes, a significant advantage of the food‐based dietary strategies employed in the DIREM trial is their potential for greater cost‐effectiveness and real‐world accessibility compared to formula‐based total diet replacement (TDR) programs. The food‐based intervention presented here may offer a more scalable and sustainable model for widespread implementation in diverse clinical and socio‐economic contexts, potentially increasing equity in access to diabetes remission strategies.

The DIREM study possesses notable strengths, including its food‐based nutritional intervention, which enhances real‐world practicality and feasibility. Our three‐arm design (control, CCR, IFCCR) extends beyond typical trials comparing single interventions against usual care, enabling direct evaluation of intermittent fasting versus continuous calorie restriction. Key limitations include the inability to blind clinicians or dietitians to group allocation due to the nature of dietary interventions, though outcome assessors and statisticians remained masked. The small number of remission events, particularly in the control group, resulted in wide confidence intervals and limited precision. Although this low rate is clinically expected given the natural history of diabetes without intervention, it presents statistical challenges and requires cautious interpretation of the effect estimates. Additionally, delivery by a specialised multidisciplinary team may limit generalisability to settings lacking comparable resources. Because our follow‐up was only 6 months, the observed remission rates may represent temporary metabolic improvements rather than durable reversal.

## 5 Conclusion

In conclusion, this 6‐month study suggests that both the calorie‐carbohydrate restriction diet and intermittent fasting combined with calorie‐carbohydrate restriction are associated with improvements in glycemic control, weight and fat loss, and rates of remission of type 2 diabetes. Although IFCCR showed greater fat mass reduction and a numerically higher remission rate, this difference was not statistically significant. These findings should be interpreted cautiously, and further research with larger sample sizes and longer follow‐up is needed to confirm these results.

## Author Contributions

N.B. investigation, data curation, methodology, writing – original draft, writing – review and editing. A.E. conceptualisation, investigation, supervision. K.D. data curation, writing – review and editing. M.S.Y. methodology, formal analysis, writing – review and editing. S.M. and V.D. investigation, data curation. S.K.R. and M.A. conceptualisation, data curation. S.E. investigation, methodology. S.S.‐B. conceptualisation, investigation, methodology, supervision, project administration, funding acquisition, writing – review and editing.

## Funding

This study was approved and funded by a grant from Tehran University of Medical Sciences (TUMS), Tehran, Iran (grant number: 1403‐2‐212‐72606).

## Ethics Statement

This study was conducted according to the principles of the Declaration of Helsinki. Ethical approval was obtained from the Tehran University of Medical Sciences Research Ethics Committee (IR.TUMS.SHARIATI.REC.1402.128).

## Consent

All participants signed informed consent forms.

## Conflicts of Interest

The authors declare no conflicts of interest.

## Supporting information


**Table S1:** Baseline characteristics in 113 participants.
**Table S2:** Key outcome variables at 3‐ and 6‐month assessments (based on per‐protocol analysis).
**Figure S1:** Glycaemia outcomes (A) changes in glycated haemoglobin (HbA1c) during the study periods (B) proportion of participants who had diabetes remission (defined as HbA1c < 6.5% [< 48 mmol/mol] and no medications for 3 months) (based on per‐protocol analysis).
**Figure S2:** Weight and body composition.

## Data Availability

The data that support the findings of this study are available from the corresponding author upon reasonable request.
