# Supplementary material for: The Effect of Integrated Lifestyle Intervention Incorporating Calorie‐Carbohydrate Restriction With or Without Time‐Restricted Feeding for Remission of Type 2 Diabetes (DIREM): A Single Blind Randomised Controlled Trial
Source: Endocrinol Diabetes Metab. 2026 Apr 11;9(3):e70209. doi: 10.1002/edm2.70209 (PMC13069986; doi:10.1002/edm2.70209)
Supplement: Supplementary file 1 — Table S1: Baseline characteristics in 113 participants. Table S2: Key outcome variables at 3‐ and 6‐month assessments (based on per‐protocol analysis). Figure S1: Glycaemia outcomes (A) changes in glycated haemoglobin (HbA1c) during the study periods (B) proportion of participants who had diabetes remission (defined as HbA1c < 6.5% [< 48 mmol/mol] and no medications for 3 months) (based on per‐protocol analysis). Figure S2: Weight and body composition. [file EDM2-9-e70209-s001.docx]

**The effect of integrated lifestyle intervention incorporating calorie-carbohydrate restriction with or without time-restricted feeding for remission of type 2 diabetes (DIREM): a single blind randomized controlled trial**

| Variables | **CCR group**  **(n=38)** | **IFCCR group**  **(n=38)** | **Control group**  **(n=37)** |
| --- | --- | --- | --- |
| Female | 19 (50.0%) | 19 (50.0%) | 18 (48.6%) |
| Male | 19 (50.0%) | 19 (50.0%) | 19 (51.4%) |
| Age, years | 49.1 (6.8) | 49.7 (7.4) | 51.4 (7.3) |
| Education status |  |  |  |
| High school or less | 3 (7.9%) | 8 (21.1%) | 6 (16.2%) |
| Diploma | 9 (23.7%) | 12 (31.6%) | 10 (27.0%) |
| University degree or higher | 26 (68.4%) | 18 (47.4%) | 21 (56.8%) |
| Employment status |  |  |  |
| Employed | 25 (65.8%) | 22 (57.9%) | 19 (51.4%) |
| Unemployed | 9 (23.7%) | 10 (26.3%) | 10 (27.0%) |
| Retired | 4 (10.5%) | 6 (15.8%) | 8 (21.6%) |
| Bodyweight, kg | 86.0 (9.9) | 86.6 (9.2) | 86.5 (12.2) |
| BMI, kg/m^2^ | 31.0 (3.1) | 31.6 (3.1) | 30.8 (2.4) |
| Waist circumference, cm | 106.3 (8.6) | 107.1 (8.6) | 109 (8.5) |
| Fat mass, kg | 29.3 (8.0) | 30.3 (8.6) | 28.2 (6.1) |
| Fat free mass, kg | 56.4 (9.2) | 56.3 (10.5) | 58.1 (12.2) |
| Duration of diabetes, years | 3.0 (1.8) | 2.8 (1.8) | 3.3 (1.8) |
| Number of diabetes medications | 4.0 (1.8) | 3.9 (1.8) | 3.8 (1.8) |
| Type of diabetes medication |  |  |  |
| Metformin | 38 (100%) | 38 (100%) | 37 (100%) |
| Sulfonylurea | 15 (39.5%) | 15 (39.5%) | 11 (29.7%) |
| DPP-4 inhibitor | 15 (39.5%) | 17 (44.7%) | 14 (37.8%) |
| Thiazolidinedione | 1 (2.6%) | 0 (0.0%) | 1 (2.7%) |
| SGLT2 inhibitor | 14 (36.8%) | 13 (34.2%) | 16 (43.2%) |
| HbA_1C%_ | 7.0 (0.6) | 7.3 (0.6) | 7.4 (1.2) |
| HbA_1C_, mmol/mol | 54.8 (43.8) | 57.6 (59.8) | 58.7 (70.6) |
| FBG, mmol/L | 7.3 (1.2) | 7.4 (1.2) | 7.5 (1.8) |
| QUCKI | 0.3 (0.00) | 0.3 (0.00) | 0.3 (0.00) |
| HOMAIR | 4.0 (1.8) | 4.4 (1.8) | 4.0 (2.4) |
| Systolic blood pressure, mm Hg | 132.6 (12.9) | 128.2 (11.1) | 127 (8.5) |
| Diastolic blood pressure, mm Hg | 87.3 (8.6) | 87.1 (9.2) | 84.8 (7.9) |
| Prescribed antihypertensive medication | 14 (36.8%) | 14 (36.8%) | 13 (35.1%) |
| Prescribed statins | 36 (94.7%) | 34 (89.5%) | 34 (91.9%) |
| Total cholesterol, mmol/L | 4.0 (0.6) | 3.8 (0.6) | 4.0 (0.6) |
| HDL cholesterol, mmol/L | 1.0 (0.2) | 1.0 (0.2) | 1.1 (0.2) |
| LDL cholesterol, mmol/L | 2.3 (0.6) | 2.1 (0.6) | 2.2 (0.6) |
| Triglycerides, mmol/L | 1.7 (1.2-2.1) | 1.6 (1.2-2.1) | 1.3 (0.9-2.1) |
| AST, U/L | 29.1 (6.2) | 29.5 (9.2) | 25.8 (6.1) |
| ALT, U/L | 35.6 (10.5) | 37.5 (17.3) | 31.6 (9.1) |
| eGFR, mL/min per 1.73 m^2^ ^*^ | 82.2 (11.1) | 80.2 (10.5) | 84.4 (9.7) |
| Current smoker | 9 (23.7%) | 7 (18.4%) | 5 (13.5%) |
| Physical activity, Met.min/week | 646.9 (125.8) | 631.1 (131.3) | 617 (115.0) |
| EQ-5D scale score | 0.54 (0.2) | 0.55 (0.2) | 0.53 (0.2) |
| Total energy intake Kcal/day | 2355.8 (298.4) | 2280.4 (304.5) | 2361.8 (350.4) |
| Carbohydrate intake g/day | 372.7 (57.3) | 362.8 (56.7) | 363.8 (66.3) |
| Protein intake g/day | 78.2 (17.9) | 75.5 (16.6) | 80.7 (10.9) |
| Total fat intake g/day | 62.7 (5.5) | 61.0 (6.8) | 65.0 (19.1) |

**Supplementary Table 1: Baseline characteristics in 113 participants**

Data are n (%), mean (SD) and median (IQR). BMI= Body Mass Index. HbA1c=glycated hemoglobin. FBG= Fasting Blood Glucose. QUICKI= Quantitative Insulin Sensitivity Index. HOMAIR= Homeostatic Model Assessment for Insulin Resistance. HDL= High Density Lipoprotein. LDL= Low Density Lipoprotein. AST= Aspartate Aminotransferase. ALT= Alanine Transaminase. eGFR= Estimated glomerular filtration rate. *According to the Modification of Diet in Renal Disease Study equation. EQ-5D=EuroQol 5 Dimensions.

**Supplementary Table 2. Key outcomes variables at 3- and 6-months assessments (based on per-protocol analysis)**

| Variables | **Baseline** | | | **3 Months** | | | | | | **6 Months** | | | | | |
| --- | --- | --- | --- | --- | --- | --- | --- | --- | --- | --- | --- | --- | --- | --- | --- |
|  | Control | CCR | IFCCR | Control | CCR | | IFCCR | | | Control | CCR | | IFCCR | | |
|  | Mean  (SE) | Mean  (SE) | Mean  (SE) | Mean  (SE) | Mean  (SE) | P^a^ | Mean  (SE) | P^b^ | P^c^ | Mean  (SE) | Mean  (SE) | P^a^ | Mean  (SE) | P^b^ | P^c^ |
| HbA1c% | 7.37  (0.15) | 7.03  (0.14) | 7.25  (0.14) | 7.24  (0.08) | 6.54  (0.07) | <0.001 | 6.41  (0.07) | <0.001 | 0.235 | 7.22  (0.10) | 6.39  (0.09) | <0.001 | 6.34  (0.09) | <0.001 | 0.747 |
| HbA1c mmol/mol | 58.75  (1.59) | 54.99  (1.56) | 57.36  (1.57) | 57.29  (0.84) | 49.63  (0.82) | <0.001 | 48.24  (0.82) | <0.001 | 0.235 | 57.03  (1.04) | 47.94  (1.03) | <0.001 | 47.47  (1.02) | <0.001 | 0.747 |
| FBG mmol/L | 7.49  (0.25) | 7.30  (0.25) | 7.36  (0.25) | 7.43  (0.14) | 6.23  (0.13) | <0.001 | 6.10  (0.13) | <0.001 | 0.493 | 7.36  (0.15) | 5.75  (0.15) | <0.001 | 5.84  (0.15) | <0.001 | 0.683 |
| QUICKI | 0.32  (0.004) | 0.32  (0.004) | 0.31  (0.004) | 0.31  (0.002) | 0.34  (0.002) | <0.001 | 0.34  (0.002) | <0.001 | 0.590 | 0.31  (0.003) | 0.34  (0.003) | <0.001 | 0.34  (0.003) | <0.001 | 0.726 |
| HOMAIR | 4.06  (0.34) | 4.05  (0.34) | 4.34  (0.34) | 4.65  (0.16) | 2.75  (0.16) | <0.001 | 2.55  (0.16) | <0.001 | 0.385 | 4.96  (0.19) | 2.43  (0.18) | <0.001 | 2.18  (0.18) | <0.001 | 0.345 |
| Weight, kg | 87.68  (1.02) | 86.24  (1.00) | 85.16  (1.00) | 86.57  (0.41) | 82.04  (0.40) | <0.001 | 81.17  (0.40) | <0.001 | 0.126 | 86.77  (0.48) | 81.24  (0.46) | <0.001 | 80.41  (0.47) | <0.001 | 0.210 |
| WC, cm | 109.89  (0.85) | 106.73  (0.83) | 105.82  (0.84) | 108.63  (0.54) | 102.82  (0.52) | <0.001 | 102.88  (0.53) | <0.001 | 0.942 | 109.07  (1.05) | 101.25  (0.99) | <0.001 | 99.89  (1.01) | <0.001 | 0.335 |
| Fat mass, kg | 29.01  (0.64) | 29.60  (0.62) | 29.24  (0.63) | 29.56  (0.46) | 24.95  (0.46) | <0.001 | 23.31  (0.46) | <0.001 | 0.011 | 29.77  (0.54) | 23.90  (0.53) | <0.001 | 22.30  (0.53) | <0.001 | 0.034 |
| Fat free mass, kg | 58.57  (1.03) | 56.33  (1.00) | 55.94  (1.01) | 56.92  (0.21) | 57.26  (0.20) | 0.241 | 57.62  (0.20) | 0.018 | 0.217 | 56.90  (0.22) | 57.56  (0.22) | 0.037 | 57.72  (0.22) | 0.010 | 0.606 |
| TC, mmol/L | 3.99  (0.13) | 4.03  (0.13) | 3.86  (0.13) | 4.21  (0.08) | 3.55  (0.08) | <0.001 | 3.58  (0.08) | <0.001 | 0.796 | 4.42  (0.08) | 3.45  (0.08) | <0.001 | 3.46  (0.08) | <0.001 | 0.943 |
| HDL, mmol/L | 1.11  (0.04) | 1.05  (0.04) | 1.06  (0.04) | 1.01  (0.02) | 1.16  (0.02) | <0.001 | 1.16  (0.02) | <0.001 | 0.980 | 0.97  (0.02) | 1.22  (0.02) | <0.001 | 1.20  (0.02) | <0.001 | 0.546 |
| LDL, mmol/L | 2.19  (0.12) | 2.25  (0.12) | 2.08  (0.12) | 2.54  (0.06) | 1.88  (0.06) | <0.001 | 1.86  (0.06) | <0.001 | 0.822 | 2.77  (0.08) | 1.77  (0.08) | <0.001 | 1.77  (0.08) | <0.001 | 0.993 |
| Triglyceride, mmol/L | 1.72  (0.13) | 1.74  (0.13) | 1.71  (0.13) | 1.90  (0.07) | 1.38  (0.07) | <0.001 | 1.28  (0.07) | <0.001 | 0.299 | 1.94  (0.08) | 1.37  (0.08) | <0.001 | 1.22  (0.08) | <0.001 | 0.189 |
| AST (U/L) | 26.15  (1.22) | 28.98  (1.20) | 29.34  (1.20) | 29.11  (0.63) | 22.48  (0.61) | <0.001 | 22.57  (0.61) | <0.001 | 0.916 | 30.36  (0.72) | 20.22  (0.70) | <0.001 | 20.70  (0.70) | <0.001 | 0.630 |
| ALT (U/L) | 32.45  (1.99) | 35.03  (1.95) | 37.27  (1.95) | 35.13  (1.11) | 27.30  (1.09) | <0.001 | 26.68  (1.10) | <0.001 | 0.685 | 36.99  (1.13) | 24.25  (1.10) | <0.001 | 23.94  (1.11) | <0.001 | 0.841 |
| Number of diabetes medications | 3.77  (0.30) | 4.07  (0.29) | 3.86  (0.29) | 4.31  (0.21) | 2.42  (0.21) | <0.001 | 2.38  (0.21) | <0.001 | 0.899 | 4.17  (0.21) | 2.42  (0.21) | <0.001 | 2.39  (0.21) | <0.001 | 0.903 |
| Systolic BP, mmHg | 126.82  (1.81) | 132.98  (1.77) | 127.96  (1.78) | 129.15  (0.88) | 120.82  (0.88) | <0.001 | 121.32  (0.86) | <0.001 | 0.692 | 129.95  (0.94) | 117.67  (0.93) | <0.001 | 117.59  (0.92) | <0.001 | 0.948 |
| Diastolic BP, mmHg | 84.98  (1.42) | 87.21  (1.39) | 87.01  (1.40) | 87.58  (0.66) | 78.86  (0.64) | <0.001 | 78.91  (0.65) | <0.001 | 0.962 | 88.24  (0.71) | 76.33  (0.69) | <0.001 | 75.33  (0.69) | <0.001 | 0.303 |
| Quality  of life* | 0.54  (0.03) | 0.54  (0.03) | 0.54  (0.03) | 0.58  (0.02) | 0.80  (0.02) | <0.001 | 0.79  (0.02) | <0.001 | 0.782 | 0.58  (0.02) | 0.85  (0.02) | <0.001 | 0.83  (0.02) | <0.001 | 0.506 |
| Information score^†^ | 3.02  (0.12) | 2.75  (0.11) | 2.89  (0.11) | 3.47  (0.25) | 4.60  (0.25) | 0.002 | 4.66  (0.25) | 0.001 | 0.872 | 3.47  (0.26) | 4.62  (0.25) | 0.002 | 4.63  (0.25) | 0.002 | 0.997 |
| Motivation score^†^ | 27.02  (0.67) | 24.12  (0.66) | 25.73  (0.66) | 23.14  (0.68) | 35.21  (0.67) | <0.001 | 36.42  (0.66) | <0.001 | 0.202 | 22.93  (0.70) | 34.51  (0.69) | <0.001 | 35.65  (0.68) | <0.001 | 0.241 |
| Behavioral skills score^†^ | 4.11  (0.14) | 3.98  (0.13) | 4.07  (0.14) | 4.11  (0.37) | 6.69  (0.37) | <0.001 | 7.02  (0.37) | <0.001 | 0.534 | 4.11  (0.37) | 6.32  (0.37) | <0.001 | 6.73  (0.37) | <0.001 | 0.428 |
| Total score^†^ | 33.57  (0.96) | 30.81  (0.94) | 32.64  (0.95) | 29.79  (0.82) | 46.50  (0.81) | <0.001 | 47.88  (0.80) | <0.001 | 0.229 | 30.14  (0.89) | 45.21  (0.87) | <0.001 | 46.99  (0.85) | <0.001 | 0.145 |

Adjusted means for outcome variables controlling for age, gender, baseline BMI, and baseline values as fixed effects. P^a^: P value comparing the mean change of response variables in CCR group vs. control group. P^b^: P value comparing the mean change of response variables in IFCCR group vs. control group. P^c^: P value comparing the mean change of response variables in IFCCR group vs. CCR group. HbA1c=glycated hemoglobin. FBG= Fasting Blood Glucose. QUICKI= Quantitative Insulin Sensitivity Index. HOMAIR= Homeostatic Model Assessment for Insulin Resistance. WC= Waist Circumstance. TC= Total Cholesterol. HDL= High Density Lipoprotein. LDL= Low Density Lipoprotein. AST= Aspartate Aminotransferase. ALT= Alanine Transaminase. SBP= Systolic blood pressure. DBP= Diastolic blood pressure. *As measured by the EuroQol 5 Dimensions scale. ** Based on Information, motivation and behavioral skills (IMB) model.

**Supplementary Figure 1.** Glycaemia outcomes (A) changes in glycated hemoglobin (HbA1C) during the study periods (B) Proportion of participants who had diabetes remission (defined as HbA1c <6.5% [<48 mmol/mol] and no medications for 3 months) (based on per-protocol analysis).

**Supplementary Figure 2.** Weight and body composition. Bodyweight (A) and fat mass (B) during the study. (C) Proportion of participants achieving weight loss targets over 6 months (based on per-protocol analysis).
